# Oncogenic *NRAS* Primes Primary Acute Myeloid Leukemia Cells for Differentiation

**DOI:** 10.1371/journal.pone.0123181

**Published:** 2015-04-22

**Authors:** Cornelia Brendel, Sabine Teichler, Axel Millahn, Thorsten Stiewe, Michael Krause, Kathleen Stabla, Petra Ross, Minh Huynh, Thomas Illmer, Marco Mernberger, Christina Barckhausen, Andreas Neubauer

**Affiliations:** 1 Department of Hematology, Oncology and Immunology, Philipps University of Marburg, and University Clinic Giessen and Marburg, Marburg, Germany; 2 Molecular Oncology, Philipps University of Marburg, Marburg, Germany; 3 Medical Clinic I, University Clinic of Technical University Dresden, Dresden, Germany; Emory University, UNITED STATES

## Abstract

*RAS* mutations are frequently found among acute myeloid leukemia patients (AML), generating a constitutively active signaling protein changing cellular proliferation, differentiation and apoptosis. We have previously shown that treatment of AML patients with high-dose cytarabine is preferentially beneficial for those harboring oncogenic RAS. On the basis of a murine AML cell culture model, we ascribed this effect to a RAS-driven, p53-dependent induction of differentiation. Hence, in this study we sought to confirm the correlation between *RAS* status and differentiation of primary blasts obtained from AML patients. The gene expression signature of AML blasts with oncogenic *NRAS* indeed corresponded to a more mature profile compared to blasts with wildtype *RAS*, as demonstrated by gene set enrichment analysis (GSEA) and real-time PCR analysis of myeloid ecotropic viral integration site 1 homolog (*MEIS1*) in a unique cohort of AML patients. In addition, *in vitro* cell culture experiments with established cell lines and a second set of primary AML cells showed that oncogenic *NRAS* mutations predisposed cells to cytarabine (AraC) driven differentiation. Taken together, our findings show that AML with inv(16) and *NRAS* mutation have a differentiation gene signature, supporting the notion that *NRAS* mutation may predispose leukemic cells to AraC induced differentiation. We therefore suggest that promotion of differentiation pathways by specific genetic alterations could explain the superior treatment outcome after therapy in some AML patient subgroups. Whether a differentiation gene expression status may generally predict for a superior treatment outcome in AML needs to be addressed in future studies.

## Introduction

12% to 19% of acute myeloid leukemia patients carry a gain-of-function mutation within the *RAS* genes [1,2,3,4], while *NRAS* is most frequently affected [5,6]. In addition, signaling of the RAS cascade is frequently enhanced by aberrations of other players of this pathway, even when *RAS* genes are not mutated [7,8,9,10]. Although oncogenic RAS is often involved in leukemic transformation of a susceptible progenitor cell, RAS can also promote differentiation in hematopoietic cells [11,12,13,14,15].

Common AML treatment regimens include the nucleoside analog cytarabine (AraC, 1β-arabinofuranosylcytosine). AraC incorporation into DNA during S phase gives rise to a replication blocking lesion. As a consequence, the DNA damage response, that also involves p53, is activated [16,17]. In AML samples derived from the Cancer and Leukemia Group B 8525 study, we have previously shown that patients whose blasts harbored oncogenic *RAS* mutations benefitted most from post-induction dose-escalation of cytarabine [4]. To deeper understand the molecular basis for this phenomenon, we took advantage of a murine MLL-ENL driven mouse model where we demonstrated a synergism between mutant (mt) RAS and AraC with regard to myeloid differentiation. This effect was p53 dependent and accompanied by a stronger, AraC provoked DNA damage response of mtRAS compared to wildtype (wt) RAS cells [17].

In order to ask if these findings [17] were also active in primary AML cells, we first sought to perform gene expression analysis. Until now, a unique gene expression pattern associated with oncogenic *RAS* mutations has not been described in AML. This may be due to the genetic heterogeneity of this disease as several genetic aberrations have been described in AML [18] which translate into a distinct biology and prognosis in case of inv(16). On this well-defined genetic background of a core binding factor (CBF) leukemia with good prognosis, we analyzed gene expression in an AML cohort positive for inversion inv(16) or *CBFB-MYH11* (cohort 1), which is frequently associated with oncogenic *RAS* mutations [5,19].

We here show that indeed oncogenic *NRAS* is associated with a more differentiated gene expression signature in human inv(16) AML. Secondly, we analyzed whether AraC and oncogenic *NRAS* cooperate in promoting differentiation. To this end, we used myeloid cell lines as well as a second cohort of 22 primary AML cases (cohort 2). AML cells with oncogenic *NRAS* mutations showed more pronounced differentiation after AraC treatment *ex vivo*.

## Patients, Materials and Methods

### Patient information and isolation of mononuclear cells

Two different AML patient cohorts were used in this study (Table 1). Samples from the Study Alliance Leukemia study group (SAL), Dresden, Germany (cohort 1): AML patients enrolled into the AML2003 trial that were positive for inversion inv(16) or positive for the PCR transcript *CBFB-MYH11*. The *CBFB-MYH11* fusion gene/transcript was detected by classical cytogenetic analysis or PCR [20]. The study was approved by the ethics committees of the University of Dresden and participating centers in agreement with the Helsinki Declaration and registered with ClinicalTrials.gov (NCT00180102, AML 2003-Standard-Therapy vs. Intensified Therapy for Adult Acute Myeloid Leukemia Patients under 60 Years) [21]. Written informed consent was obtained from each patient [21]. Mononuclear cells were isolated from bone marrow (BM) of 34 AML patients with inversion16 with/without *NRAS*12/13, 61 or *KRAS* mutation S1 Table) by density gradient centrifugation using Ficoll-Hypaque (1.077 g/mL).

Samples from Marburg (cohort 2): Mononuclear cells were isolated from BM or peripheral blood (PB) of 22 untreated AML patients (S2 Table) by density gradient centrifugation. The patients gave written informed consent.

**Table 1:** AML patient cohorts used in this study.

**AML cohort**	**Characteristics**	**Number**	**Material**	**Source**	**Purpose**
**1**	inversion 16 karyotype; wt *RAS* / mt*NRAS*	34	RNA	Dresden, Germany (included in clinical trial)	cDNA array (n = 34), GSEA (n = 31), *MEIS1* qPCR (n = 31)
**2**	irrespective of karyotype; wt *RAS* / mt*NRAS*	22	vital cells	Marburg, Germany (not included in a clinical trial)	*in vitro* cell culture and differentiation analysis by flow cytometry (n = 22)

GSEA: gene set enrichment analysis

### Analysis of *RAS* status

Samples from Dresden (cohort 1): high-performance liquid chromatography based method or peptide nucleic acid-mediated PCR clamping as described previously [6,22,23]. Samples from Marburg (cohort 2): high resolution melting (HRM) PCR with SYTO9 (Invitrogen) on a rotor-gene 6000 device (Corbett Research) [24]. For more details see S1 MaterialsMethods.

### Microarray and GSEA

Microarray-based expression analysis was performed as described previously [25] with 34 AML patient samples with inversion 16 karyotype with/without *NRAS*12/13, 61 or *KRAS*12/13 mutation (cohort 1). MIAME compliant microarray data are available in the ArrayExpress database (www.ebi.ac.uk/arrayexpress, accession number E-MTAB-2090). GSEA was conducted only with mt*NRAS* (n = 12, S1 Table) and wt*RAS* samples (n = 19, S1 Table) using standard parametrization and the GSEA software (version 2.0.10) with a weighted signal-to-noise metric. As reference gene set database, curated gene sets derived from the Molecular Signature Database. More information about enriched gene sets can be found in: Ivanova et al. 2002; Croonquist et al. 2003; Jaatinen et al. 2006; Ben-Porath et al. 2008 [26,27,28,29]. For more details see S1 Materials and Methods.

### Quantitative real-time PCR for MEIS1 expression

PCR for *MEIS1* was performed under following conditions: 95°C 15 min; then 45 cycles of 94°C 15 sec, 60°C 30 sec, 72°C 30 sec. Primer sequences: *GAPDH*-for: 5’-CTCCTCCAC CTTTGACGCTG-3’, *GAPDH*-rev: 5’-ACCACCCTGTTGCTGTAGCC-3’. *MEIS1*-for: 5’-ACGG CATCTACTCGTTCAGG-3’, *MEIS1-*rev: 5’-GTTGTCCAAGCCATCACCTT-3’. GAPDH served as internal reference gene. For details see S1 Materials and Methods.

### Morphology analysis of cytospin slides

The AML cell lines HL-60 (mt*NRAS*) and U937 (wt*RAS*; both purchased from German Collection of Microorganisms and Cell Cultures) were seeded at a density of 5x10^4^/ml. After incubation with 100 nM or 350 nM AraC (controls with corresponding amount of DMSO) for 48h, cytospin slides (2x10^4^ cells/spot) were prepared and stained according to the May-Grünwald-Giemsa method.

### Quantitative real-time PCR for *CD14* expression

PCR of U937 and HL-60 cells was performed under following conditions: 95°C 15 min; then 45 cycles of 94°C 15 sec, 57°C 30 sec, 72°C 30 sec. Primer sequences: *GAPDH*: see *MEIS1* PCR. *CD14*-for: 5’-GTTCGGAAGACTTATCGACCA T-3’, *CD14-*rev: 5’-ACAAGGTTCTGGCGTGGT-3’. For more details see S1 Materials and Methods.

### Analysis of and *FLT3* status

5ng of DNA of cohort 2 were used for PCR with fluorophore-labeled primers in a Biorad T100 thermal cycler. Primer sequences originate from Kiyoi et al., 1999 [30]. PCR program: 95°C 5 min; 30 cycles of 94°C 30 sec, 57°C 30 sec, 72°C 1 min; then 60°C 45 min. PCR products were analyzed on an ABI PRISM 310 genetic analyzer with GeneScan analysis software.

### In vitro cell culture and analysis of differentiation of primary AML cells by flow cytometry

Primary AML blasts (irrespective of karyotype, cohort 2, S2 Table) obtained from frozen PB or BM were seeded on a mesenchymal stem cell (MSC) layer. For enrichment of MSC, spongiform bone fragments were obtained from hip replacement surgery. The procedures were approved by the ethics committee at Philipps University Marburg (study no. 64/01 and 25/10) and patients gave written informed consent. For more information, see Brendel et al., 2005 [31]. Primary AML blasts were cultivated in RPMI-1640 medium, containing 20% FCS and 1% Penicillin/Streptomycin at 37°C and 5% CO_2_ in a humidified atmosphere.

After 24h, medium was changed, AraC was added and 48h later cells were harvested. Concomitant with the actual samples, HL-60 cells (oncogenic *NRAS*) were treated with AraC and analyzed for differentiation by flow cytometry in the same manner and used as positive control (S3 Table).

Samples were incubated with the following antibodies for 20 min at 4°C in PBS selected according to patients differentiation marker profile: CD45-PerCP-Cy5.5 (2D1, BD, #332784), CD11c–PE (B-ly6, BD, #333149), CD15-FITC (HI98, BD, #332778), CD34-PE (8G12, BD, #345802); HLA-DR-FITC (B8.12.2, Beckman Coulter, #PNIMO463U), CD14-PE (M5E2, BD, #345785) or CD117-APC (104D2, BD, #333233). DNA was counterstained with 4',6-Diamidinophenyl-indole (DAPI) before analysis on an LSRII cytometer (BD Biosciences). Results were analyzed independently in a blinded manner with FLOWJO 9.6.4 or FACS-DIVA 6.0 software. Myeloblast/immature leukemias (FAB class M0, M1, M2) were considered as „differentiating (positive)“, if either CD15 or CD11c increased, or HLA-DR or CD34 decreased. Monocytic leukemias (FAB class M4, M5) were considered positive for differentiation when CD15 declined, CD14, HLA-DR or CD11c increased, because immature monocytes often express CD15 highly and CD14 weekly. HLA-DR is strongly expressed on immature blasts and on mature monocytes. CD15 is weekly expressed on immature monocytes but strongly expressed in mature granulocytes [32]. For more details see S1 Materials and Methods.

### Statistics

Mann Whitney test and student’s t-test were calculated by Prism software (Graphpad Software, Inc.), Fisher’s exact test via http://www.quantitativeskills.com/sisa/statistics/fisher.htm.

## Results

### Gene expression pattern of AML patients with inversion 16 and oncogenic *RAS* mutations

We were interested whether our previous observation [17] that oncogenic RAS is linked to a more differentiated phenotype also holds true for primary human AML blasts. To this end, we performed a GSEA in order to highlight potential differentiation-associated pathways. The 31 treatment-naïve AML patients samples (cohort 1, Table 1 and S1 Table) analyzed by GSEA harbored an inversion 16 karyotype associated with a *CBFB-MYH11* fusion gene or expressed a *CBFB-MYH11* fusion transcript. 12 of 31 AML patients additionally carried oncogenic *NRAS* (12/13 or 61) mutations.

**Fig 1:**
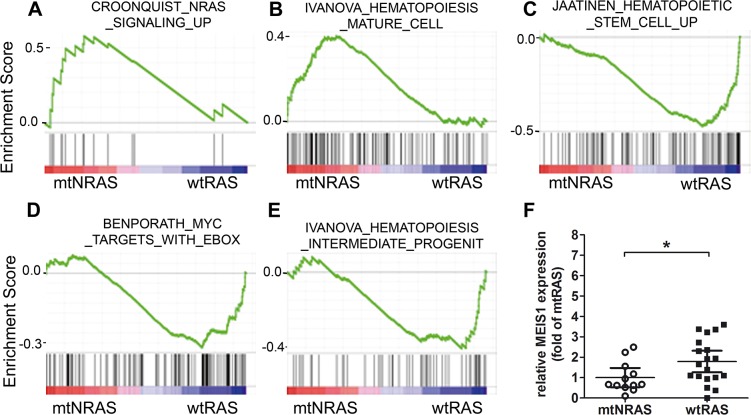
Impact of *NRAS* status on the transcriptome of primary AML blasts. (A-E) Enrichment plots as obtained by GSEA software for the gene sets CROONQUIST_NRAS_SIGNALING_UP, positive control; IVANOVA HEMATO-POIESIS_MATURE_CELL; JAATINEN_HEMATOPOIETIC_STEM _CELL _UP; BENPORATH_MYC_TARGETS_WITH_EBOX; IVANOVA_HEMATOPOIESIS_INTER-MEDIATE_PROGENITOR. Primary AML blasts harboring wt*NRAS* were compared with mt*NRAS* (12/13 or 61) blasts. (F) Relative expression of *MEIS1* in primary AML blasts carrying wt*RAS* or mt*NRAS* as examined by real-time PCR. The graph shows the median with 95% confidence interval. Results were normalized to *MEIS1* expression of mt*NRAS* blasts. *GAPDH* expression served as internal control. *: p = 0.025 (Mann Whitney test).

Among the 50 most strongly enriched gene sets for each genotype (wt or mt*NRAS*, S5 and S6 Tables), we found several differentiation- or stemness-associated gene sets (Fig 1). Fig 1A shows the enrichment of mt*NRAS* regulated genes in mt*NRAS* samples, which corroborates the validity of our data. A gene set representing differentiation and maturation of hematopoietic cells was found to be enriched in mt*NRAS* samples (Fig 1B), while immaturity-associated gene sets, including a gene set representing an association with the stem cell factor MYC, were enriched in the wt*NRAS* cohort (Fig 1C–1E).

### Expression of the homeobox gene *MEIS1* in AML inversion 16 patient samples with wt*RAS*/mt*NRAS*

Homeobox (HOX) genes, among which is *MEIS1*, are typically downregulated during hematopoietic stem cell differentiation and maturation and are considered as stemness markers [33]. This prompted us to measure *MEIS1* expression by quantitative real-time PCR in 31 samples of primary AML blasts obtained from patients with inversion 16 (cohort 1). 12 patients harbored oncogenic *NRAS*, 19 patients carried w*tRAS* genes. Indeed, *MEIS1* expression was significantly higher (1.9-fold) in blasts with wt*RAS* than in mt*NRAS* blasts (Fig 1F).

### Oncogenic *NRAS* together with cytarabine drives AML cells into myeloid differentiation *in vitro*

**Fig 2:**
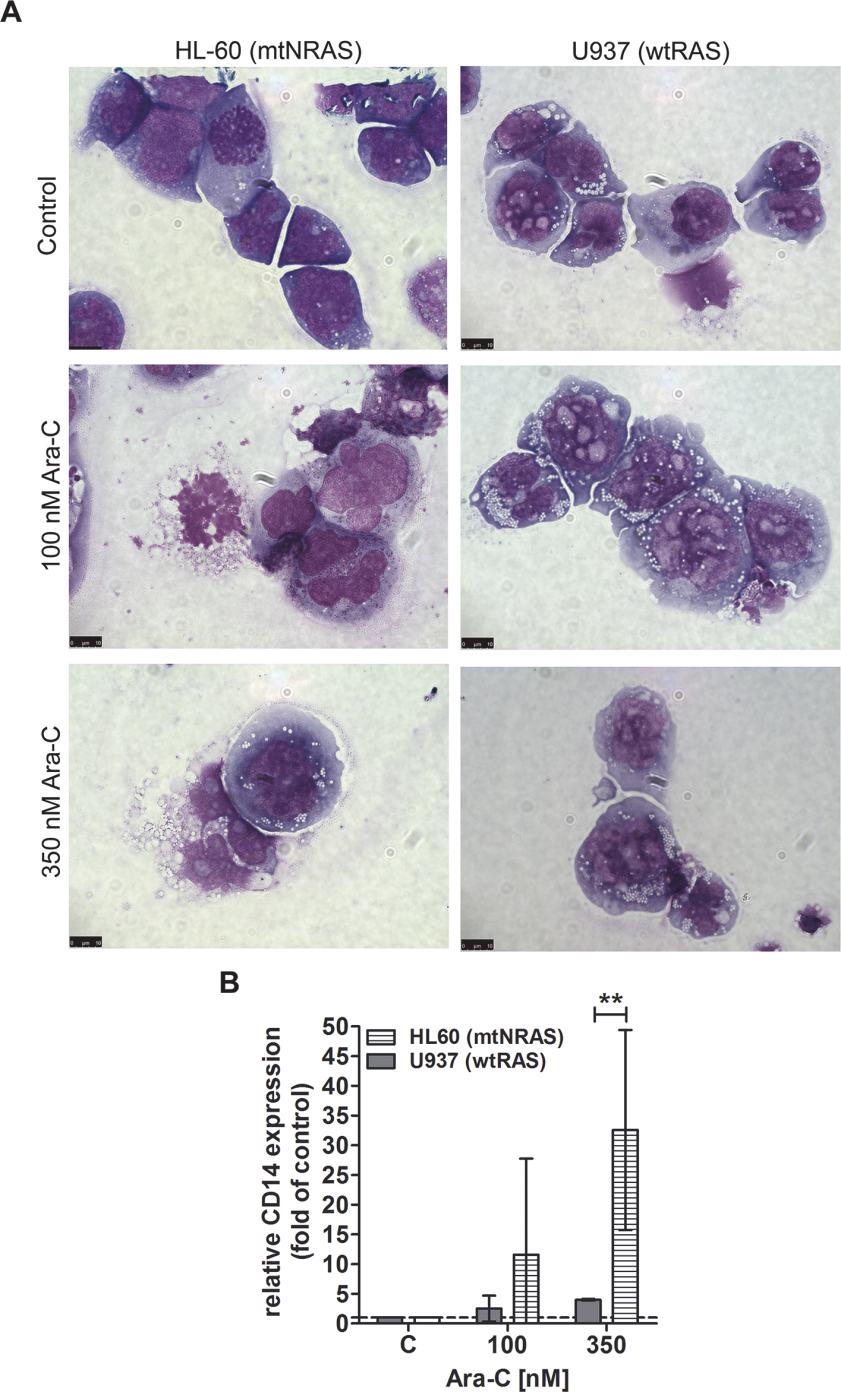
AraC induces differentiation in the mt*NRAS* harboring AML cell line HL-60. (A) May-Grünwald-Giemsa staining of HL-60 and U937 (wt*RAS*) cells 48h after AraC treatment at indicated doses. (B) *CD14* expression in HL-60 and U937 cells 48 h after AraC-treatment at indicated doses as determined by quantitative real-time PCR. *GAPDH* was used for normalization. The graph shows the median with 95% confidence interval. Results were normalized to the respective control of each cell line. **: p = 0.002 (Student’s t-test).

**Fig 3:**
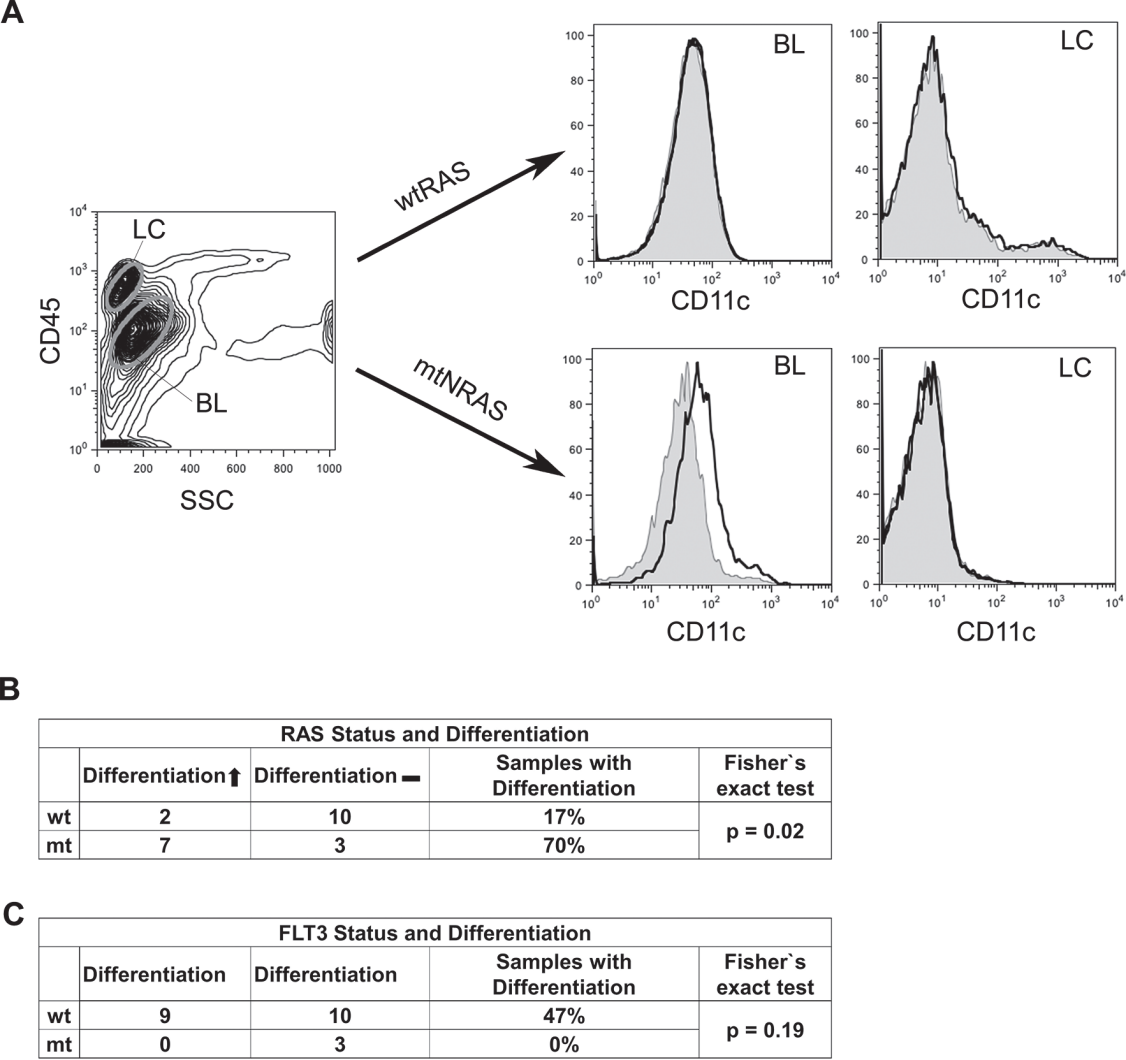
AraC induces differentiation predominantly in primary AML blasts with oncogenic *NRAS*. (A) Left panel: Example of gating for lymphocytes (LC) and AML blasts (BL) according to SSC/CD45 signals after live gating. wt*RAS* panels: CD11c expression of wt*RAS* AML blasts (left) and LC (right) in untreated samples (solid grey curve) or samples treated with 100 nM AraC (black line). mt*RAS* panels: CD11c expression of mt*NRAS*12/13 blasts (left) and LC (right) treated as indicated above. (B) Summary of the *in vitro* responses to AraC treatment in terms of differentiation of 22 primary AML blasts with or without *NRAS* mutation. *Samples with differentiation* describes the portion of samples with differentiation response (diff. ↑) to AraC in relation to all samples in the wt*RAS* or mt*NRAS* cohort, respectively. Fisher`s exact test: p = 0.02. (C) Summary of the *in vitro* responses to AraC treatment in terms of differentiation of 22 primary AML blasts with or without FLT3-ITD. Fisher`s exact test: p = 0.19.

On the basis of the gene expression data, we sought to substantiate our observation by comparing the differentiation response of wt*RAS* and mt*NRAS* cells to AraC treatment. Fig 2A shows the morphological differentiation response provoked by AraC treatment in the two AML cell lines HL-60 (mtN*RAS*) and U937 (wt*RAS*). While brightening and granulation of the cytoplasm indicate differentiation of HL-60 cells after AraC treatment, this effect could not be observed in U937 cells. The morphological changes of HL-60 cells were accompanied by increased expression (32.6-fold) of the differentiation marker *CD14* (Fig 2B). In order to investigate if AraC also provokes differentiation in primary mt*RAS* blasts, we used 22 samples from AML patients with or without *NRAS* mutation (cohort 2; S2 Table) and treated them *in vitro* with AraC. The cells were subsequently examined by flow cytometry by probing for a patient specific selection of the markers CD11c, CD15, CD14, CD117, CD34 or HLA-DR, which are frequently used to assess differentiation of myeloid cells. Fig 3A represents a sample of the wt*RAS* and the mt*NRAS* cohort, while the left panel exemplifies gating for blasts (BL) and lymphocytes (LC). The analyzed wt*RAS* blasts (upper 2 panels) were obtained from a treatment-naïve, 77-year-old Caucasian man suffering from AML type FAB M2 (patient 5, S2 and S4 Tables). The expression of CD11c remained constant on AML BL under AraC treatment, which was interpreted as a non-differentiation response. Analysis of CD11c expression on LC served as internal control and was not affected. The results obtained with primary AML blasts received from a treatment-naïve 57-year-old Caucasian woman diagnosed with AML type FAB M2 and harboring an *NRAS*12/13 mutation (lower 2 panels; patient 17, S2 and S4 Tables) provide a representative example of an *in vitro* differentiation response of the mt*NRAS* cohort, reflected in an increase of CD11c expression. In summary, 7 of 10 (70%) mt*NRAS* samples displayed an AraC-triggered change of at least one differentiation-indicating marker, whereas only 2 of 12 (17%) wt*RAS* samples did (Fig 3B, histograms of all patients see S4 Table). FLT3-ITD (Internal tandem duplication) mutation is not accompanied by an induction of differentiation (Fig 3C). In this patient cohort, *NRAS* status significantly correlated with *in vitro* differentiation upon AraC treatment.

## Discussion

The *RAS* genes encode for a highly conserved group of small GTPase proteins. RAS is physiologically activated upon ligand binding to receptors such as receptor tyrosine kinases. The ligands, and the cellular context, dictate the cellular outcome after activation of the RAS pathway. While gain-of-function mutations of *RAS* represent one of the most abundant aberrations in human cancer, their prognostic significance for AML patients remains controversial. Some studies have reported that mutated *RAS* has a negative or no impact on clinical outcome of AML patients, whereas others have described a positive influence of oncogenic RAS [19,30,34,35,36,37,38,39]. Potential reasons for the inconsistent conclusions could be the consideration of different clinical parameters such as patient age or genetic heterogeneity. In addition, the treatment may be of major influence on the outcome, depending on the oncogenic background of the respective AML.

In previous studies we and others have shown that oncogenic RAS dictates poor risk in AML patients treated with low dose AraC [4], while it is beneficial when patients are treated with high dose AraC (HiDAC) [4,36] with respect to relapse risk or overall survival. Several reports suggested that oncogenic RAS itself triggers the DNA damage response by provoking replication stress [40,41]. In line with the activation of the DNA damage response by oncogenic RAS, we found that the addition of DNA-damaging or replication stress-inducing agents to cells expressing oncogenic RAS drives a p53-dependent, DNA-damage induced myeloid differentiation. It is conceivable that excessive replication stress resulting from high-dose AraC treatment and replication stress induced by oncogenic RAS cooperate in promoting differentiation of AML blasts.

For the present study, we sought to analyze whether the previous observations that oncogenic RAS is associated with more pronounced differentiation holds also true in primary AML cells employing whole genome gene expression analysis and GSEA of AML patients included in a multicenter trial (AML2003) [21]. In order to minimize confounders of different underlying genetic lesions, only patients with a specific genetic aberration, namely inversion 16, were selected. Indeed, oncogenic *NRAS* was associated with a specific expression pattern, and among the top 50 gene sets (S5 and S6 Tables), several sets of stemness or differentiation genes could be found. This is, to the best of our knowledge, the first analysis showing a specific gene expression pattern (i.e. differentiation) associated with *RAS* mutations in AML. This finding is corroborated by Shen et al., who discovered a role for RAS in myeloid differentiation [12,13].

If oncogenic RAS is associated with differentiation, we next sought to study whether this effect could be enhanced by additional replication stress, for instance caused by AraC. Our data show that AraC induces myeloid differentiation in terms of morphology and protein surface expression preferentially in mt*NRAS* cells. Interestingly, internal tandem duplications of the FLT3 gene, also known to activate the MAPK signaling pathway, did not cause the same effect on differentiation. We conclude that AML cells with oncogenic *NRAS* are more prone to AraC-driven differentiation as compared to AML cells with wildtype *NRAS*, and that the *NRAS* mutation is the pivotal lesion for this effect.

Therefore, depending on the cellular context where it is expressed, oncogenic RAS may trigger differentiation that is even more enhanced when cells are exposed to the replication blocking agent AraC. We therefore suggest that induction of myeloid differentiation may be a rational target of chemotherapy that has previously not been appreciated in AML other than acute promyelocytic leukemia (APL).

## Supporting Information

S1 Materials and Methods Supporting Information about Materials and Methods. (PDF)

S1 Table Characteristics of 34 AML Patients of Cohort 1. Patients carried inv(16) and wt*RAS* or mt*RAS* and samples were used for cDNA array; GSEA and *MEIS1* analysis using quantitative real time PCR.
(PDF)

S2 Table Characteristics of 22 AML Patients of Cohort 2. Samples were obtained before treatment of the patient and used for *in vitro* cell culture and analysis of differentiation by flow cytometry.
(PDF)

S3 Table Representative HL-60 Flow Cytometry Histograms of *in vitro* Differentiation Experiments. HL-60 positive control, exemplifying live gating and differentiation response. An HL-60 sample was carried along with each experiment with patients’ samples. The occurrence of the HL-60 response in terms of cell death and differentiation induction by AraC confirmed successful performance of the experiment.
(PDF)

S4 Table Representative Patients’ Samples Flow Cytometry Histograms of *ex vivo* Differentiation Experiments. For differentiation positive samples, the differentiation indicating marker is depicted. For differentiation negative samples (i.e. no shift with any marker observable), one of the analyzed markers is shown representatively for all markers.
(PDF)

S5 Table List of Top 50 Gene Sets Showing Enrichment in the mt*NRAS* Cohort. (PDF)

S6 Table List of Top 50 Gene Sets Showing Enrichment in the wt*NRAS* Cohort. (PDF)

S7 Table Correlation Between *NPM1* Status and Differentiation. (PDF)
