# Supplementary material for: Oncogenic NRAS Primes Primary Acute Myeloid Leukemia Cells for Differentiation
Source: PLoS One. 2015 Apr 22;10(4):e0123181. doi: 10.1371/journal.pone.0123181 (PMC4406710; doi:10.1371/journal.pone.0123181)
Supplement: S1 Materials and Methods — (PDF) [file pone.0123181.s001.pdf]

Supporting Materials and Methods

Analysis of RAS status

Samples from Marburg (cohort1): Genomic DNA was extracted by applying the QIAamp DNA minikit (Qiagen) according to the manufacturer's instructions. PCR was performed under following conditions: 95°C 2 min; then 40 cycles of 95°C 15 sec, annealing temperature 15 sec, 72°C 15 sec; one cycle of 95°C 1 sec, 72°C 90 sec. Ramp from 72 °C to 95 °C rising at 0.2°C per sec. Primer sequences: *NRAS*61-for: 5'-CACACCCCCAGGATTCTTAC-3', *NRAS*61-rev: 5'-TGGCAAATACACAGAGGAAGC-3, annealing temperature 60.6°C. *NRAS*12/13-for: 5'-CAGGTTCTTGCTGGTGTGAA-3', *NRAS*12/13-rev: 5'-CACTGGGCCTCACCTCTATG-3', annealing temperature 58.8°C.

Microarray and GSEA

Total RNA was isolated from primary blasts of 34 AML samples with inversion 16 karyotype with or without additional NRAS12/13, 61 or KRAS mutation (cohort 1; QuickPrep mRNA purification kit, Pharmacia Biotech).

Probes were collapsed to gene level by selecting the maximum expression value of the probe set. Genes were ranked in descending order based on real value using the weighted signal-to-noise metric (p=1). Nominal p values were calculated based on permutation tests using phenotype permutation with 1000 permutations without balancing. Normalized enrichment scores were calculated as described in Subramanian et al., 2005 [1] and gene sets were ranked accordingly. Gene sets including less than 15 genes or more than 500 genes were discarded.

Reverse transcription and quantitative real-time PCR for *MEIS1*

Cohort 1: 300ng of total RNA were transcribed into c(omplementary) DNA (Omniscript RT Kit, Qiagen).

Cohort 2: 1µg of total RNA was transcribed into cDNA (Omniscript RT Kit, Qiagen). cDNA was diluted 1:5 in water before use.

PCR was conducted with the QuantiTect SYBR Green PCR kit (Qiagen) on a quantitative real-time PCR device (ABI): Each PCR plate was equipped with a U937 reference sample (German Collection of Microorganisms and Cell Cultures) to allow for comparison of different plates. Samples were analyzed in duplicates. Expression data were evaluated using the ΔΔCt method in comparison to the standardized control sample from U937.

Quantitative real-time PCR for *CD14*

PCRs for CD14 of HL-60 and U937 cells were carried out with the QuantiTect SYBR Green PCR kit (Qiagen) using a Mastercycler ep realplex (Eppendorf).

Analysis of *NPM1* status

See “Analysis of FLT3 status” in main manuscript. Primer sequences were taken from Thiede et al., 2006 [2].

References

1. Subramanian A, Tamayo P, Mootha VK, Mukherjee S, Ebert BL, et al. (2005) Gene set enrichment analysis: a knowledge-based approach for interpreting genome-wide expression profiles. Proc Natl Acad Sci U S A 102: 15545-15550.

2. Thiede C, Koch S, Creutzig E, Steudel C, Illmer T, et al. (2006) Prevalence and prognostic impact of NPM1 mutations in 1485 adult patients with acute myeloid leukemia (AML). Blood 107: 4011-4020.
