# Supplementary material for: Oncogenic NRAS Primes Primary Acute Myeloid Leukemia Cells for Differentiation
Source: PLoS One. 2015 Apr 22;10(4):e0123181. doi: 10.1371/journal.pone.0123181 (PMC4406710; doi:10.1371/journal.pone.0123181)
Supplement: S1 Table — Patients carried inv(16) and wtRAS or mtRAS and samples were used for cDNA array; GSEA and MEIS1 analysis using quantitative real time PCR. (PDF) [file pone.0123181.s002.pdf]

Table S1. Characteristics of 34 AML Patients of Cohort 1.

Pat no	karyotype	material	blasts [%]	RAS status	used for array[a]	used for GSEA[b]	Rel. expression MEIS1 qPCR[c]
1	47,XX,inv(16)(p13q22),+22 [8]	BM	50	K-RAS 12/13 mut	x	--	--
2	46,XY,inv(16)(p13;q22) [30]	BM	63,5	wt	x	x	0,14711
3	47,XY,+13,inv(16)(p13q22) [5]	BM	72	N-RAS 12/13 mut	x	x	0,03874
4	46,XY,inv(16)(p13q22) [9]	BM	73	wt	x	x	0,10331
5	inv(16)	BM	73,5	wt	x	x	0,20950
6	46,XY,t(1;4)(q25;q12),inv(16)(p13q22) [20	BM	40	wt	x	x	0,08717
7	47,XY,inv(16)(p13q22),+22 [4]	BM	65,5	wt	x	x	0,06911
8	46,XY,inv(16)(p13q22) [13]	BM	48,5	wt	x	x	0,14359
9	47,XY,inv(16)(p13q22) ,+22 [2]	BM	57,5	K-RAS 12/13 mut	x	--	--
10	47,XY,+8,inv(16)(p13q22) [19]	BM	70,5	wt	x	x	0,06337
11	46,,XY,-6,del(7)(q?22),inv(16)(p13q22),+mar,inc [1]	BM	86	wt	x	x	0,17016
12	46,XY,inv(16)(p13q22) [17]	BM	65	N-RAS 61 mut	x	x	0,02469
13	47,XY,inv(16)(p13q?22),+22 [9]	BM	80	wt	x	x	0,05872
14	47,XX,inv(16)(p13q22),+22 [4]	BM	36,5	N-RAS 61 mut	x	x	0,13966
15	46,XY,inv(16)(p13q22)	BM	71	K-RAS 12/13 mut	x	--	--
16	47,XY,t(16;16),+22 [3]	BM	56,5	N-RAS 12/13 mut	x	x	0,09706
17	47,XY,inv(16)(p13q22),+22 [5]	BM	40	wt	x	x	0,11991
18	48,XX,inv(16)(p13q22),+add(23),+22	BM	50	wt	x	x	0,03158
19	46,XY,inv(16)(p13q22) [17]	BM	70	wt	x	x	0,02328
20	47,XY,inv(16)(p13q22),+22 [20]	BM	50	N-RAS 12/13 mut	x	x	0,04181
21	46,XX,inv(16)(p13q22) [13]	BM	54	N-RAS 12/13 mut	x	x	0,05913
22	47,XY,inv(16)(p13q22),+8 [5]	BM	46,5	N-RAS 61 mut	x	x	0,03901
23	46,XY,inv(16)(p13q22) [14]	BM	48	N-RAS 61 mut	x	x	0,00619
24	46,XY,t(1;17)(q25;q21),inv(16)(p13q22) [27	BM	73	wt	x	x	0,21095
25	46,XX,t(7;16)(q11;p11-13) [22]	BM	64	wt	x	x	0,20096
26	46,XY,inv(16)(p13q22) [19]	BM	81,5	wt	x	x	0,06887
27	47,XY,inv(16)(p13q22),+21][17]	BM	53	N-RAS 61 mut	x	x	0,03246
28	46,XX,inv(16)(p13q22),+22 [7]	BM	67	N-RAS 12/13 mut	x	x	0,07356
29	46,XY,inv(16)(p13q22) [17]	BM	82,5	wt	x	x	0,06675
30	inv(16)	BM	38	wt	x	x	0,00003
31	46,XX,t(16;16)(q22;p13.1) [19]	BM	83	wt	x	x	0,22453
32	47,XX,+22,?inv(16)(p13q22),?del(16)(q22q24) [cp20]	BM	65	N-RAS 12/13 mut	x	x	0,15550
33	46,XY,inv(16)(p13q22) [11]	BM	62	N-RAS 12/13 mut	x	x	0,04123
34	46,XX,?inv(16)(p13q22) [5]; 50,XX,+9,+?13,+14,?inv(16)(p13q22),+22 [8]	BM	67	wt	x	x	0,12116

Patients carried inv(16) and wtRAS or mtRAS and samples were used for cDNA array; GSEA and *MEIS1* analysis using quantitative real time PCR.

[a]see www.ebi.ac.uk/arrayexpress, accession number E-MTAB-2090

[b]see figure 1A-E

[c]see Figure 1F

--: not used/not analyzed
