# Supplementary material for: Oncogenic NRAS Primes Primary Acute Myeloid Leukemia Cells for Differentiation
Source: PLoS One. 2015 Apr 22;10(4):e0123181. doi: 10.1371/journal.pone.0123181 (PMC4406710; doi:10.1371/journal.pone.0123181)
Supplement: S2 Table — Samples were obtained before treatment of the patient and used for in vitro cell culture and analysis of differentiation by flow cytometry. (PDF) [file pone.0123181.s003.pdf]

Table S2. Characteristics of 22 AML Patients of Cohort 2.

Pat no	Age (y)	Sex	[a]FAB	Karyotype	Source	Blasts [%]	RAS status	FLT3 (ITD[d])	NPM1 status	Used for differentiation analysis	Differentiation response and indicating marker
1	50	f	M4	46,XX	BM[b]	63	wt	wt	mt	x	positive according to CD11c signal
2	71	m	M4	46,XY (20)	BM	70	wt	wt	mt	x	negative; no diff. indicating change of HLA-DR, CD11c, CD14, CD15, CD34, CD45
3	45	m	-	44,XY, -3,dic (5;7), (q12;q11), +8, der (12)t(12;17)(p12;q21) ,-17 (5) 44,XY,-3,dic (5;7) (q12;q11), +8, der (12)t(12;17) (p12;q21),del (16)(q13),der (17) t(16;17) (?;q11) t(5;16)(?;?) t(5;17) (?;q21),-17 (9) 46,XY (6)	BM	20	wt	wt	wt	x	negative; no diff. indicating change of HLA-DR, CD11c, CD14, CD15, CD34, CD45
4	60	m	M1	46,XY [25]	BM	78	wt	mt	mt	x	negative; no diff. indicating change of HLA-DR, CD11c, CD14, CD15, CD34, CD45
5	77	m	M2	46,XY	BM	70	wt	wt	mt	x	negative; no diff. indicating change of HLA-DR, CD11c, CD14, CD15, CD34, CD45
6	58	f	M4	46,XX [11]	PB	5	wt	wt	mt	x	negative; no diff. indicating change of HLA-DR, CD11c, CD14, CD15, CD34, CD45
7	24	m	M0	46,XY,del(6)(q13q23) [3]	BM	82	wt	wt	wt	x	negative; no diff. indicating change of HLA-DR, CD11c, CD14, CD15, CD34, CD45
8	70	m	-	46,XY	PB[c]	83	wt	wt	wt	x	negative; no diff. indicating change of HLA-DR, CD11c, CD14, CD15, CD34, CD45
9	71	f	M2	t(8;21)	PB	82	wt	wt	wt	x	negative; no diff. indicating change of HLA-DR, CD11c, CD14, CD15, CD34, CD45
10	74	m	M4	45,X,-Y [20]	PB	66	wt	mt	mt	x	negative; no diff. indicating change of HLA-DR, CD11c, CD14, CD15, CD34, CD45
11	47	m	M4	46,XY	BM	90	wt	wt	mt	x	negative; no diff. indicating change of HLA-DR, CD11c, CD14, CD15, CD34, CD45
12	33	f	M5a	46, XX.ish	PB	90	wt	wt	wt	x	positive; CD11c

				der(19)ins(19;11)(p13;q23q23)(MLL5+) [20]							
13	71	m	-	45,XY,t(3;3)(q21;q26),-7 [8] 46, XY [2]	PB	17	N-RAS 12/13 mt	wt	wt	x	positive; CD15
14	75	f	-	MDS/AML	BM		N-RAS 12/13 mt	wt	wt	x	negative; no diff. indicating change of HLA-DR, CD11c, CD14, CD15, CD34, CD45
15	41	f	M1	46,XX [23]	BM	65	N-RAS 12/13 mt	wt	wt	x	positive; CD15
16	36	f	M4	46,XX, inv(3) [6],45XX,-5,add(12), frgl. t(5;12)01/06: 46, XX, inv(3)(q21q26)[1],/44, Idem,der(5)t(5;16)(q13,q21), der(12)t(12;15)(p12;q21)-15,-16[11]/46,Idem,t(12;22)(p12;q12)[8]. nuc ish 3q26 (EVI prox x 2,EVI1 dist x 3)(EVI1 prox sep EVI1 dist x 0)	PB	30	N-RAS 61 mt	wt	wt	x	positive; HLA-DR
17	57	f	M2	47,XX, +4 [4]; 46 XX [20]	BM	17	N-RAS 12/13 mt	wt	mt	x	positive; CD11c
18	73	m	M4	45,X,-Y[20]	BM	37	N-RAS 61 mt	wt	wt	x	positive; CD15
19	57	m	M2	46,XY,t(10;11)(p12;q23),der(13)t(13;17)(q34;q12) [18] 52,XY,+4,+6,+8,t(10;11)(p12;q23),der(13)t(13;17)(q34;q12),+der(13)t(13;17)(q34;q12),+21,+22 [2]	BM	86	N-RAS 61 mt	wt	wt	x	negative; no diff. indicating change of HLA-DR, CD11c, CD14, CD15, CD34, CD45
20	71	f	M2	46,XX	BM	90	N-RAS 61 mt	mt	mt	x	negative; no diff. indicating change of HLA-DR, CD11c, CD14, CD15, CD34, CD45
21	75	f	M3	t(15;17)	PB	98	N-RAS 61 mt	wt	wt	x	positive; CD14
22	56	m	M2	46,XY [20]	BM	85	N-RAS 12/13 mt	wt	wt	x	positive; HLA-DR

Samples were obtained before treatment of the patient and used for *in vitro* cell culture and analysis of differentiation by flow cytometry.

[a]french-american-british

[b]bone marrow

[c]peripheral blood

[d]internal tandem duplication
