# Supplementary material for: Oncogenic NRAS Primes Primary Acute Myeloid Leukemia Cells for Differentiation
Source: PLoS One. 2015 Apr 22;10(4):e0123181. doi: 10.1371/journal.pone.0123181 (PMC4406710; doi:10.1371/journal.pone.0123181)
Supplement: S3 Table — HL-60 positive control, exemplifying live gating and differentiation response. An HL-60 sample was carried along with each experiment with patients’ samples. The occurrence of the HL-60 response in terms of cell death and differentiation induction by AraC confirmed successful performance of the experiment. (PDF) [file pone.0123181.s004.pdf]

Table S3. Representative HL-60 Flow Cytometry Histograms of *in vitro* Differentiation Experiments.

HL-60			
**Live Gating**			**Analysis of Differentiation Marker**
**0 nM AraC**	**100 nM AraC**	**350 nM AraC**	**0, 100, 350 nM AraC[a]**
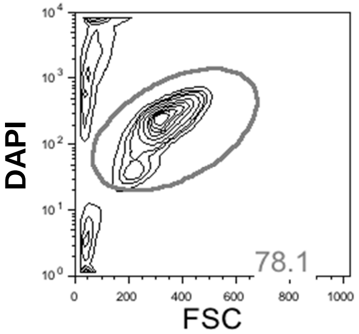	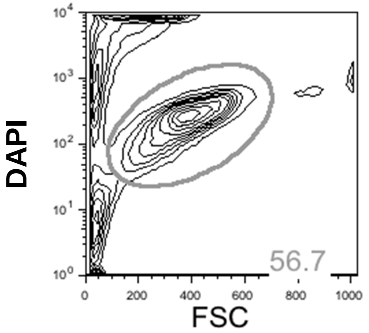	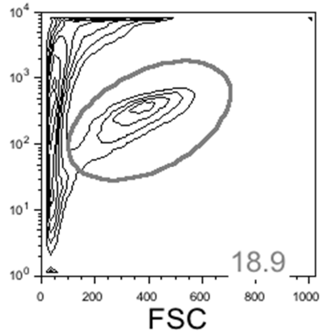	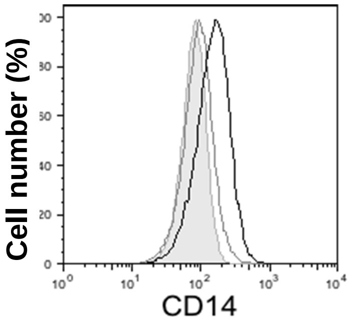

HL-60 positive control, exemplifying live gating and differentiation response. An HL-60 sample was carried along with each experiment with patients' samples. The occurrence of the HL-60 response in terms of cell death and differentiation induction by AraC confirmed successful performance of the experiment. [a]grey filled curve: 0 nM, grey curve 100 nM, black curve 350 nM AraC
