# Supplementary material for: Oncogenic NRAS Primes Primary Acute Myeloid Leukemia Cells for Differentiation
Source: PLoS One. 2015 Apr 22;10(4):e0123181. doi: 10.1371/journal.pone.0123181 (PMC4406710; doi:10.1371/journal.pone.0123181)
Supplement: S4 Table — For differentiation positive samples, the differentiation indicating marker is depicted. For differentiation negative samples (i.e. no shift with any marker observable), one of the analyzed markers is shown representatively for all markers. (PDF) [file pone.0123181.s005.pdf]

**Table S4. Representative Patients' Samples Flow Cytometry Histograms of *in vitro* Differentiation Experiments**.

| Patient | 1 (wt RAS) | 2 (wt RAS) | 3 (wt RAS) | 4 (wt RAS) | 5 (wt RAS) |
|---|---|---|---|---|---|
| Lymphocytes | 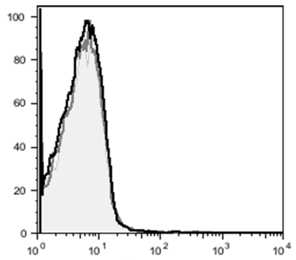 | 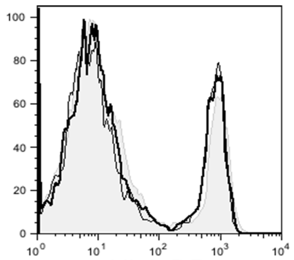 | 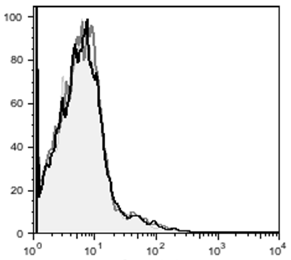 | 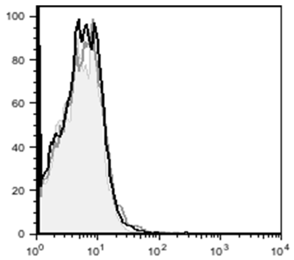 | 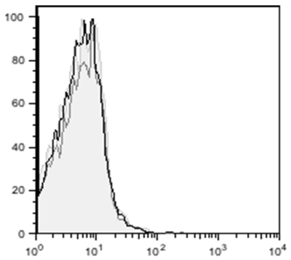 |
| Blasts | 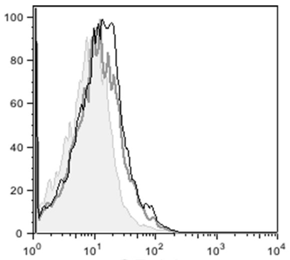 | 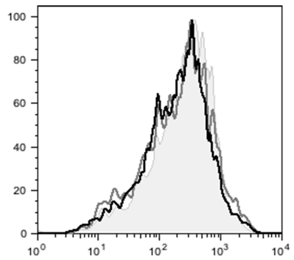 | 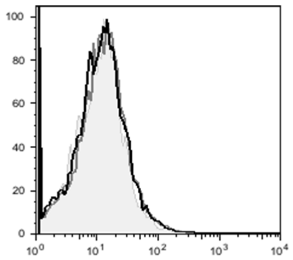 | 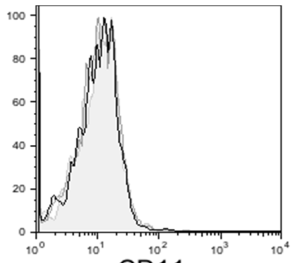 | 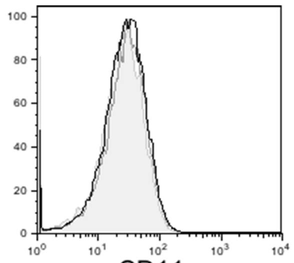 |
| Differen-tiation | positive | negative | negative | negative | negative |

| Patient | 6 (wt RAS) | 7 (wt RAS) | 8 (wt RAS) | 9 (wt RAS) | 10 (wt RAS) |
|---|---|---|---|---|---|
| **Lymphocytes** | 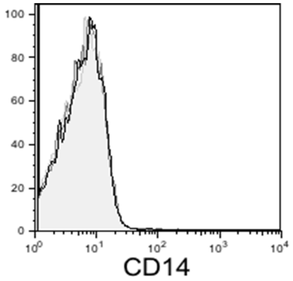 | 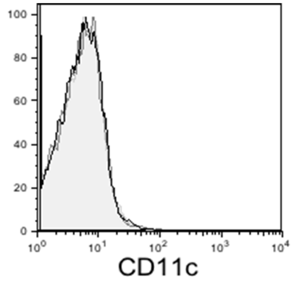 | 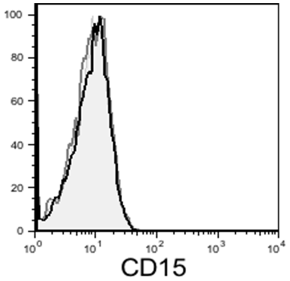 | 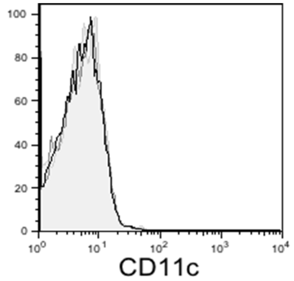 | 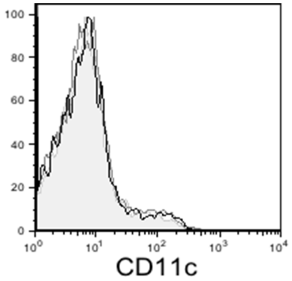 |
| **Blasts** | 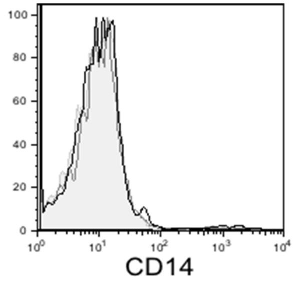 | 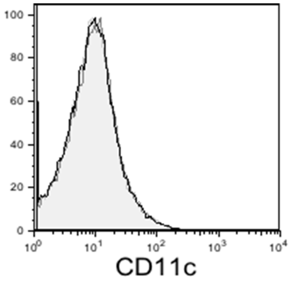 | 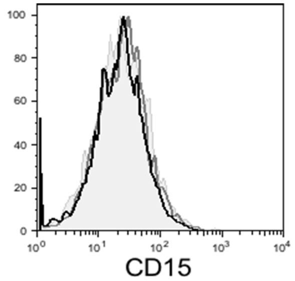 | 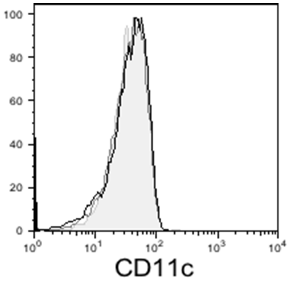 | 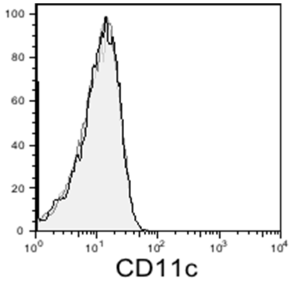 |
| **Differen-tiation** | **negative** | **negative** | **negative** | **negative** | **negative** |

| Patient | 11 (wt RAS) | 12 (wt RAS) | 13 (mt RAS) | 14 (mt RAS) | 15 (mt RAS) |
| --- | --- | --- | --- | --- | --- |
| Lymphocytes | 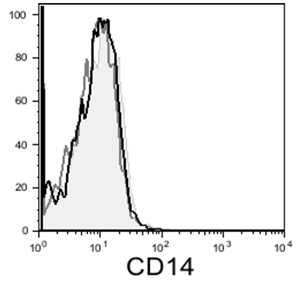  | 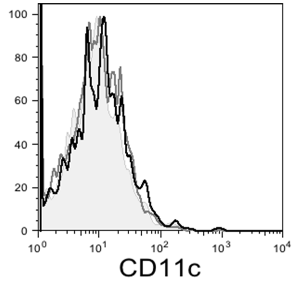  | 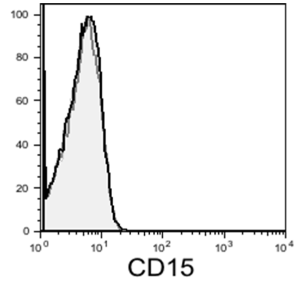  | 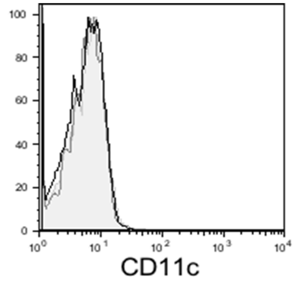  | 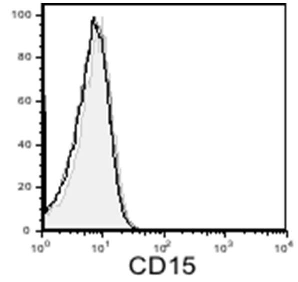  |
| Blasts | 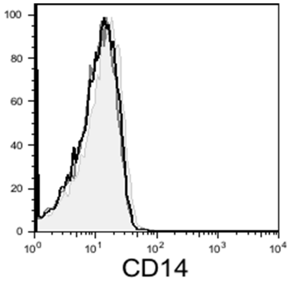  | 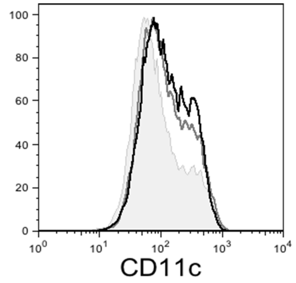  | 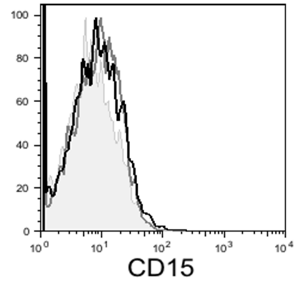  | 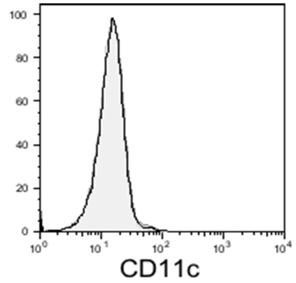  | 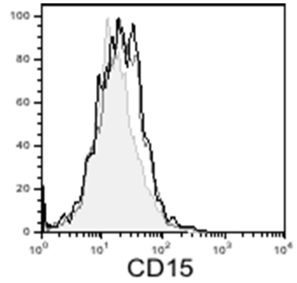  |
| Differen-tiation | negative | positive | positive | negative | positive |

| Patient | 16 (mt RAS) | 17 (mt RAS) | 18 (mt RAS) | 19 (mt RAS) | 20 (mt RAS) |
|---|---|---|---|---|---|
| Lymphocytes | 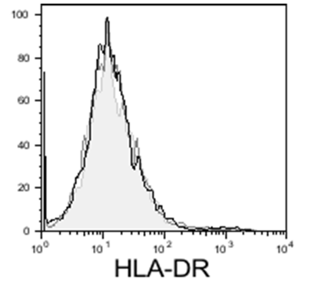  | 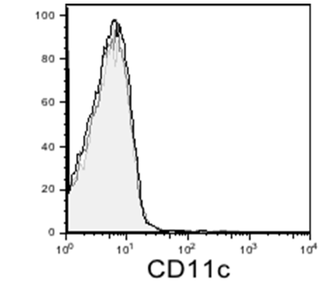  | 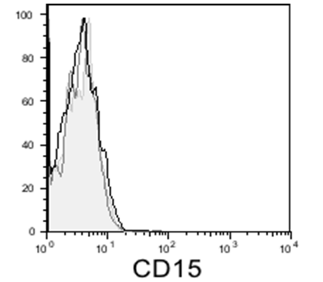  | 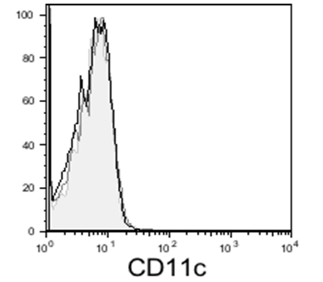  | 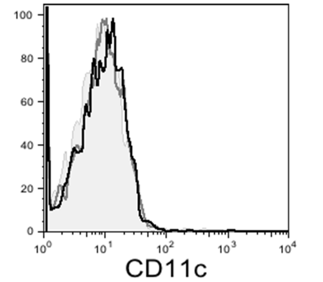  |
| Blasts | 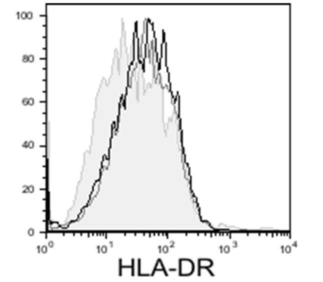  | 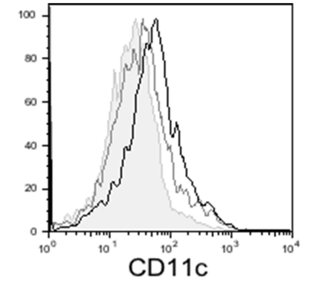  | 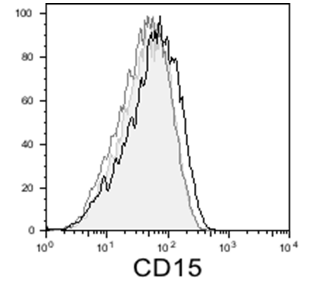  | 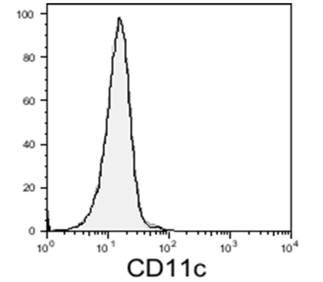  | 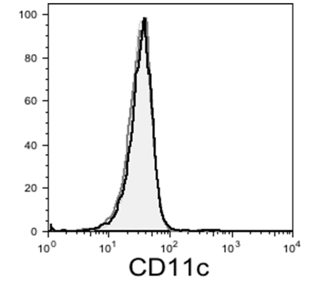  |
| Differen-tiation | positive | positive | positive | negative | negative |

| Patient | 21 (mt RAS) | 22 (mt RAS) |
|---|---|---|
| **Lymphocytes** | 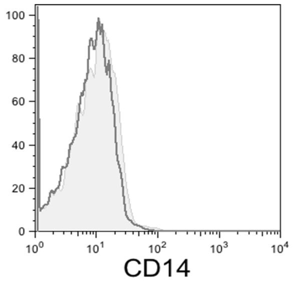 | 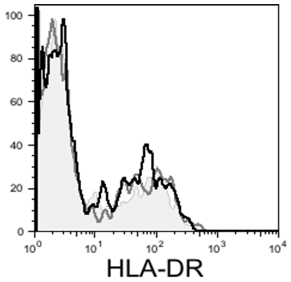 |
| **Blasts** | 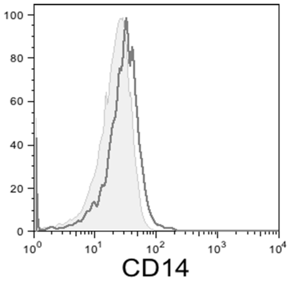 | 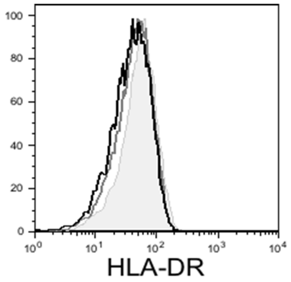 |
| **Differen-tiation** | **positive** | **positive** |

For differentiation positive samples, the differentiation indicating marker is depicted. For differentiation negative samples (i.e. no shift with any marker observable), one of the analyzed markers is shown representatively for all markers. Grey filled curve: 0 nM; grey curve 100 nM; black curve 350 nM AraC.
