# Supplementary material for: Oncogenic NRAS Primes Primary Acute Myeloid Leukemia Cells for Differentiation
Source: PLoS One. 2015 Apr 22;10(4):e0123181. doi: 10.1371/journal.pone.0123181 (PMC4406710; doi:10.1371/journal.pone.0123181)
Supplement: S5 Table — (PDF) [file pone.0123181.s006.pdf]

Table S5. List of Top 50 Gene Sets Showing Enrichment in the mtNRAS Cohort.

NO.	NAME	SIZE	FDR q-val	RANK AT MAX
1	RHEIN_ALL_GLUCOCORTICOID_THERAPY_UP	33	1.0	1290
2	YIH_RESPONSE_TO_ARSENITE_C2	15	1.0	978
3	HOFFMANN_SMALL_PRE_BII_TO_IMMATURE_B_LYMPHOCYTE_DN	21	1.0	369
4	CROMER_METASTASIS_UP	35	1.0	697
5	REACTOME_SPHINGOLIPID_METABOLISM	15	1.0	1290
6	IVANOVA_HEMATOPOIESIS_MATURE_CELL	104	1.0	1318
7	CASORELLI_APL_SECONDARY_VS_DE_NOVO_UP	16	1.0	742
8	AMIT_EGF_RESPONSE_60_HELA	25	1.0	625
9	LUI_THYROID_CANCER_CLUSTER_1	25	1.0	714
10	DAVICIONI_PAX_FOXO1_SIGNATURE_IN_ARMS_UP	26	1.0	1124
11	IKEDA_MIR1_TARGETS_UP	27	1.0	1530
12	YOSHIOKA_LIVER_CANCER_EARLY_RECURRENCE_DN	20	1.0	475
13	AMIT_EGF_RESPONSE_240_HELA	24	1.0	1274
14	LEE_LIVER_CANCER_MYC_E2F1_UP	28	1.0	453
15	SATO_SILENCED_BY_METHYLATION_IN_PANCREATIC_CANCER_2	21	1.0	1045
16	CHEOK_RESPONSE_TO_HD_MTX_DN	16	1.0	748
17	GUENTHER_GROWTH_SPHERICAL_VS_ADHERENT_DN	18	1.0	786
18	WANG_ESOPHAGUS_CANCER_VS_NORMAL_DN	53	1.0	979
19	CROONQUIST_NRAS_SIGNALING_UP	15	1.0	1057
20	CADWELL_ATG16L1_TARGETS_UP	32	1.0	1037
21	UDAYAKUMAR_MED1_TARGETS_DN	117	1.0	1078
22	KEGG_RIG_I_LIKE_RECEPTOR_SIGNALING_PATHWAY	26	1.0	960
23	TURASHVILI_BREAST_DUCTAL_CARCINOMA_VS_LOBULAR_NORMAL_DN	28	1.0	17
24	CROONQUIST_NRAS_VS_STROMAL_STIMULATION_UP	20	1.0	1319
25	DAZARD_RESPONSE_TO_UV_SCC_DN	67	1.0	1448
26	FARMER_BREAST_CANCER_APOCRINE_VS_LUMINAL	149	1.0	1171
27	BRUECKNER_TARGETS_OF_MIRLET7A3_DN	33	1.0	1080
28	REACTOME_DIABETES_PATHWAYS	59	1.0	652
29	LEE_AGING_MUSCLE_UP	23	1.0	941
30	REACTOME_CHEMOKINE_RECEPTORS_BIND_CHEMOKINES	19	1.0	814
31	HAHTOLA_MYCOSIS_FUNGOIDES_CD4_UP	34	1.0	1039
32	BOSCO_INTERFERON_INDUCED_ANTIVIRAL_MODULE	27	1.0	1119
33	STANELLE_E2F1_TARGETS	17	1.0	1504
34	LIEN_BREAST_CARCINOMA_METAPLASTIC_VS_DUCTAL_UP	31	1.0	1448
35	REACTOME_COMPLEMENT_CASCADE	16	1.0	895
36	TIEN_INTESTINE_PROBIOTICS_2HR_DN	45	1.0	1440
37	WENG_POR_TARGETS_LIVER_UP	15	1.0	1567
38	ACEVEDO_FGFR1_TARGETS_IN_PROSTATE_CANCER_MODEL_UP	100	1.0	814
39	REACTOME_LIPID_DIGESTION_MOBILIZATION_AND_TRANSPORT	16	1.0	350
40	KEGG_CYTOKINE_CYTOKINE_RECEPTOR_INTERACTION	79	1.0	890
41	INGRAM_SHH_TARGETS_DN	23	1.0	616
42	HAN_SATB1_TARGETS_DN	197	1.0	854
43	PAPASPYRIDONOS_UNSTABLE_ATEROSCLEROTIC_PLAQUE_UP	31	1.0	1284
44	PHONG_TNF_RESPONSE_NOT_VIA_P38	156	1.0	797
45	REACTOME_INNATE_IMMUNE_SYSTEM	94	1.0	895
46	HALMOS_CEBPA_TARGETS_UP	28	1.0	757
47	MCMURRAY_TP53_HRAS_COOPERATION_RESPONSE_DN	29	1.0	1693
48	LEE_LIVER_CANCER_E2F1_UP	33	1.0	802
49	STAMBOLSKY_RESPONSE_TO_VITAMIN_D3_UP	31	1.0	642
50	VART_KSHV_INFECTION_ANGIOGENIC_MARKERS_UP	65	1.0	1264
