# Supplementary material for: Oncogenic NRAS Primes Primary Acute Myeloid Leukemia Cells for Differentiation
Source: PLoS One. 2015 Apr 22;10(4):e0123181. doi: 10.1371/journal.pone.0123181 (PMC4406710; doi:10.1371/journal.pone.0123181)
Supplement: S6 Table — (PDF) [file pone.0123181.s007.pdf]

Table S6. List of Top 50 Gene Sets Showing Enrichment in the wtRAS Cohort.

NO.	NAME	SIZE	FDR q-val	RANK AT MAX
1	CHAUHAN_RESPONSE_TO_METHOXYESTRADIOL_UP	28	0.7817699	392
2	REACTOME_ANTIGEN_ACTIVATES_B_CELL_RECEPTOR_LEADING_TO_GENERATION_OF_SECOND_MESSENGERS	16	0.7319749	864
3	JAATINEN_HEMATOPOIETIC_STEM_CELL_UP	101	0.77682006	996
4	DAIRKEE_CANCER_PRONE_RESPONSE_BPA_E2	56	0.7492026	1126
5	VERHAAK_AML_WITH_NPM1_MUTATED_DN	107	0.8158842	881
6	MULLIGHAN_NPM1_MUTATED_SIGNATURE_1_DN	60	0.7217086	628
7	IRITANI_MAD1_TARGETS_DN	17	0.66001815	661
8	BARRIER_COLON_CANCER_RECURRENCE_UP	15	0.58679706	1276
9	REACTOME_MRNA_SPLICING_MINOR_PATHWAY	18	0.68668956	586
10	MULLIGHAN_MLL_SIGNATURE_2_DN	125	0.70364106	997
11	MULLIGHAN_MLL_SIGNATURE_1_DN	106	0.8441565	792
12	WAKABAYASHI_ADIPOGENESIS_PPARG_RXRA_BOUND_36HR	58	0.8577143	600
13	YAO_TEMPORAL_RESPONSE_TO_PROGESTERONE_CLUSTER_14	57	0.8253886	835
14	BIOCARTA_BCR_PATHWAY	21	0.8030949	1248
15	ZHAN_MULTIPLE_MYELOMA_CD2_DN	15	0.7697215	1121
16	KEGG_PURINE_METABOLISM	55	0.7344584	713
17	REACTOME_TRNA_AMINOACYLATION	19	0.73105586	815
18	REACTOME_CYTOSOLIC_TRNA_AMINOACYLATION	15	0.72158587	711
19	KEGG_AMINOACYL_TRNA_BIOSYNTHESIS	16	0.7975474	711
20	PARK_HSC_AND_MULTIPOTENT_PROGENITORS	24	0.7583208	1170
21	KEGG_BASE_EXCISION_REPAIR	17	0.7424035	661
22	GNATENKO_PLATELET_SIGNATURE	23	0.7426827	873
23	GAVIN_FOXP3_TARGETS_CLUSTER_T7	41	0.7731801	1772
24	MORI_EMU_MYC_LYMPHOMA_BY_ONSET_TIME_UP	39	0.7791584	842
25	CHNG_MULTIPLE_MYELOMA_HYPERPLOID_UP	24	0.8467468	559
26	CAIRO_HEPATOBLASTOMA_CLASSES_UP	256	0.8367328	1203
27	KRIGE_RESPONSE_TO_TOSEDOSTAT_24HR_DN	308	0.8841616	1379
28	REACTOME_FORMATION_OF_THE_HIV1_EARLY_ELONGATION_COMPLEX	19	0.8746315	706
29	CREIGHTON_ENDOCRINE_THERAPY_RESISTANCE_1	190	0.88252944	675
30	PENG_GLUTAMINE_DEPRIVATION_DN	161	0.95065475	562
31	MULLIGHAN_NPM1_SIGNATURE_3_DN	75	0.96014965	628
32	PRAMOONJAGO_SOX4_TARGETS_DN	25	0.9479066	708
33	HSIAO_HOUSEKEEPING_GENES	190	0.9881997	719
34	REACTOME_CHROMOSOME_MAINTENANCE	36	1.0	1077
35	PID_IL2_STAT5PATHWAY	16	1.0	1397
36	REACTOME_DNA_REPAIR	50	1.0	1088
37	JAIN_NFKB_SIGNALING	35	1.0	1124
38	RICKMAN_TUMOR_DIFFERENTIATED_WELL_VS_POORLY_UP	89	1.0	1029
39	REACTOME_MRNA_SPLICING	42	1.0	586
40	ABRAMSON_INTERACT_WITH_AIRE	16	1.0	1077
41	IVANOVA_HEMATOPOIESIS_INTERMEDIATE_PROGENITOR	51	1.0	665
42	ZHAN_MULTIPLE_MYELOMA_SUBGROUPS	21	1.0	731
43	GRAHAM_CML_QUIESCENT_VS_NORMAL_QUIESCENT_DN	22	1.0	852
44	REACTOME_INTEGRIN_ALPHAIIB_BETA3_SIGNALING	15	1.0	1058
45	BILANGES_SERUM_AND_RAPAMYCIN_SENSITIVE_GENES	22	1.0	559
46	BENPORATH_MYC_TARGETS_WITH_EBOX	113	1.0	1171
47	JOHANSSON_GLIOMAGENESIS_BY_PDGFB_UP	22	1.0	1460
48	REACTOME_INTERACTIONS_OF_VPR_WITH_HOST_CELLULAR_PROTEINS	16	1.0	1307
49	REN_BOUND_BY_E2F	33	1.0	1563
50	POTTI_CYTOXAN_SENSITIVITY	20	1.0	705
