# Supplementary material for: Oncogenic NRAS Primes Primary Acute Myeloid Leukemia Cells for Differentiation
Source: PLoS One. 2015 Apr 22;10(4):e0123181. doi: 10.1371/journal.pone.0123181 (PMC4406710; doi:10.1371/journal.pone.0123181)
Supplement: S7 Table — (PDF) [file pone.0123181.s008.pdf]

Table S7. Correlation Between *NPM1* Status and Differentiation.

NPM1 Status and Differentiation				
	Differentiation ↑	Differentiation —	Samples with Differentiation	Fisher's exact test
wt	7	6	54%	p=0.12
mt	2	7	29%	
